# Virtual Reality High-Intensity Interval Training Exergaming Compared to Traditional High-Intensity Circuit Training Among Medical Students: Pilot Crossover Study

**DOI:** 10.2196/63461

**Published:** 2025-01-07

**Authors:** Pietro Merola, Marcos Barros Cardoso, Gabriel Barreto, Matheus Carvalho Chagas, Luana Farias Oliveira Saunders, Bryan Saunders, Danilo Cortozi Berton

**Affiliations:** 1 Move Sapiens, Hospital de Clínicas de Porto Alegre, Porto Alegre, Brazil; 2 Hospital de Clínicas de Porto Alegre, Porto Alegre, Brazil; 3 Postgraduate Program in Pulmonary Sciences, Federal University of Rio Grande do Sul, Porto Alegre, Brazil; 4 Medical School, Federal University of Rio Grande do Sul, Porto Alegre, Brazil; 5 Applied Physiology and Nutrition Research Group, Faculty of Medicine, University of São Paulo, São Paulo, Brazil; 6 Biomedical Engineering, Federal University of ABC, São Bernardo do Campo, Brazil; 7 Center of Lifestyle Medicine, Faculty of Medicine, University of São Paulo, São Paulo, Brazil; 8 Applied Physiology and Nutrition Research Group, School of Physical Education and Sport and Faculdade de Medicina, Universidade de São Paulo, São Paulo, Brazil

**Keywords:** virtual reality, VR, high-intensity interval training, exercise motivation, exergame, physical activity, exercise, heart rate

## Abstract

**Background:**

This study evaluated the effectiveness of a virtual reality (VR) high-intensity interval training (HIIT) boxing protocol compared to traditional high-intensity circuit training (HICT) in improving exercise motivation, engagement, and physiological responses among 30 healthy medical students.

**Objective:**

The purpose was to compare the VR HIIT protocol, which involved using an Oculus Quest 2 for a futuristic exoskeleton game experience, with a traditional 12-exercise HICT.

**Methods:**

In total, 30 medical students engaged in both VR HIIT, using an Oculus Quest 2 for a futuristic exoskeleton game experience, and a traditional 12-exercise HICT. Metrics included heart rate (HR) and blood lactate levels before and after exercise alongside ratings of perceived exertion and the Situational Motivation Scale.

**Results:**

VR HIIT showed significantly higher mean HR (mean 161, SD 15 vs mean 144, SD 11 bpm; *d*=1.5; *P*<.001), peak HR (mean 182, SD 15 vs mean 176, SD 11 bpm; *d*=0.8; *P*=.001), and ratings of perceived exertion (mean 16, SD 2 vs mean 15, SD 2; *d*=0.4; *P*=.03). Postexercise lactate levels were higher in HICT (mean 8.8, SD 4.5 vs mean 10.6, SD 3.0 mmol/L; *d*=0.6; *P*=.006). Intrinsic motivation and other psychological measures showed no significant differences, except for lower fatigue in HICT (*d*=0.5; *P*=.02).

**Conclusions:**

VR HIIT significantly enhances physiological parameters while maintaining intrinsic motivation, making it a viable alternative to traditional HICT. However, the short-term nature of this study is a limitation, and future research should explore the long-term engagement and therapeutic impacts of VR exercise in diverse and clinical populations.

## Introduction

Exercise and physical activity contribute to enhanced health outcomes, leading to a reduction in the risk of chronic diseases such as cardiovascular disease, diabetes, and various types of cancer [1]. Despite the well-documented benefits of regular moderate- and high-intensity exercise in reducing health risk factors, a significant portion of the global population remains physically inactive. According to the World Health Organization [2], approximately 28% of adults worldwide do not engage in sufficient physical activity, as detailed in the “Global Action Plan on Physical Activity 2018‐2030” report. Physical inactivity has been identified as the fourth leading cause of mortality globally, accounting for approximately 6% of deaths, while obesity contributes to about 5% of mortality [1]. This lack of physical activity significantly increases the risk of heart disease and diabetes across various countries and social groups regardless of income level [1]. Furthermore, it has been demonstrated that physical exercise is fundamentally important for energy balance and body mass control [3] and for mental health and sleep quality [4]. One of the most cited barriers to exercising is the lack of time and motivation [5].

High-intensity interval training (HIIT) offers a solution to these barriers by providing a time-efficient and engaging exercise modality. HIIT consists of alternating short “bursts” of intense exercise with passive rest or active periods of low-intensity exercise [6,7]. Studies show that HIIT is equally beneficial, or perhaps even superior, to traditional continuous aerobic exercise in many variables related to health and fitness, such as cardiovascular endurance, metabolic rate, and muscle strength [7]. Specifically, for exercise to be considered true HIIT, it must include periods of exercise reaching 85%‐95% of the maximum heart rate (HR) during the high-intensity intervals [8].

Additionally, HIIT’s design facilitates sustaining high-intensity activity peaks during exercise sessions. The high-intensity nature of HIIT protocols means that a complete exercise session can be performed in a shorter period (7-minute exercise) compared to classic continuous or endurance exercise protocols, making it a practical solution for time-restricted individuals [8]. However, sustaining the high intensity of HIIT can be challenging and uncomfortable, which can potentially decrease motivation for individuals [9].

In recent years, exergames (a combination of video games and exercise) have been proposed as a solution to improve motivation and engagement in physical exercise practice [9,10], and it has already demonstrated that exergames can bring benefits to the physical and mental health of players of different ages [11]. For example, exergames have been shown to improve physical fitness parameters such as cardiovascular endurance and muscle strength as well as enhance mental health outcomes such as motivation, affect, and mood restoration [12,13]. The advancement of virtual reality (VR) technologies, which allow greater sensory immersion, has provided an evolution of these devices from mere entertainment tools to potential serious games with significant health benefits [14,15]. A study suggested that playing VR exergames helps to promote enhancements in mood in young adults [16]. In addition, VR games using stationary bikes have incorporated HIIT protocols, effectively achieving the intensity required for cardiovascular and metabolic benefits. Studies have shown that VR enhances performance during HIIT [14], improves motivation [5], and maintains the necessary exercise intensity for health outcomes [17].

VR boxing may be a suitable exercise activity, given that it is feasible and effective with an HIIT protocol and logistically compatible without the need for specialized equipment. Boxing has been demonstrated to be feasible in VR [18], and high-intensity boxing training has been shown to be effective for improving fitness, making it suitable for HIIT [19]. This is endorsed by a recent study, which suggests that engaging in VR fitness boxing games can lead to vigorous physical activity with high energy expenditure comparable to traditional forms of exercise [20].

To distinguish an HIIT VR exergame from existing models [20], we emphasize a design that prioritizes greater freedom of movement, enabling players to engage in high-intensity exercises without the constraints of rigid gameplay mechanics [21,22]. Unlike traditional VR games that synchronize movement to specific patterns or beats, where players must ever dodge or hit approaching orbs and objects [20], the new approach allows for dynamic and unrestricted physical activity within a clear HIIT time structure [6,23]. During each bout, players are encouraged to deliver as many shots as possible, reinforcing the all-out effort characteristic of HIIT [6], while maintaining a more immersive and intense workout experience [21,22]. In this study, we focused on the acute effects of a single session of the game to analyze exercise intensity using HR and blood lactate concentration, standard metrics in HIIT for assessing exercise intensity and physiological stress [6,23], and the immediate motivation to perform at higher intensities. This approach was chosen over a chronic study to directly assess how the game influences the intensity and motivation during high-intensity exercise sessions.

The objective was to evaluate how a VR exergame, Move Sapiens, influenced acute physiological responses and exercise motivation in comparison to a traditional high-intensity circuit training (HICT) model. The primary outcome of this study was the physiological response to VR HIIT compared to the control, a traditional HICT, measured by HR and blood lactate levels. Secondary outcomes included psychological measures such as intrinsic motivation, identified regulation, external regulation, and amotivation, assessed using the Situational Motivation Scale (SIMS). Exploratory outcomes included ratings of perceived exertion (RPE) and symptoms of simulator sickness.

## Methods

### Participants

The sample size calculation was based on the exergame study of Martin-Niedecken et al [9], which, although not identical in design, also focused on an HIIT exergame. With an effect size of *d*=0.73, guided by cardiac responses reported in their study, we aimed for 80% power and a 5% significance level. This necessitated at least 11 participants per condition or group to provide objective, quantifiable data critical for evaluating exercise intensity and effectiveness. G*Power software (Heinrich-Heine-Universität Düsseldorf) was used for this transparent and reproducible calculation. However, due to the convenience of the sample, this study increased the participant pool to 30 healthy individuals aged 18 to 30 years, consisting of both male and female medical students from the Federal University of Rio Grande do Sul School of Medicine (Table 1). Recruitment was conducted through advertising on social networks and within the university community. Participation in the research was entirely voluntary, with students given the option to freely choose whether to take part. Nonparticipation did not result in any detriment to their university activities, ensuring that the right to choose was fully respected without any prejudice or consequence. Maximal HR was calculated using the 220−age formula [24].

Participants were required to be healthy and free of major health issues, including severe psychiatric disorders, cardiovascular diseases such as congenital heart defects or arrhythmias, serious chronic conditions like uncontrolled type 1 diabetes, binocular vision anomalies, or upper and lower limb neuromuscular restrictions. Additionally, individuals with recent muscular injuries, flu-like symptoms, or any infectious conditions that could hinder HIIT performance were also excluded. Participants were classified as gamers or nongamers based on their self-reported video gaming habits. Nongamers were defined as those who reported playing less than 1 hour of video games per week over the past 2 years [25], a criterion that did not include experience with VR gaming, given that everyone reported having no previous experience with VR exergames.

**Table 1.** Demographic and physiological characteristics (N=30).

Characteristic	Values
Age (years), mean (SD)	24 (3)
Body mass (kg), mean (SD)	69.0 (11.3)
Height (m), mean (SD)	1.69 (0.09)
BMI (kg/m^2^), mean (SD)	24.1 (2.8)
Maximum heart rate (bpm), mean (SD)	196 (3)
**Sex, n (%)**	
Female	12 (40)
Male	18 (60)
Gamer, n (%)	19 (63)
Nongamer, n (%)	11 (37)
**IPAQ SF^a^, n (%)**	
High	16 (53)
Moderate	6 (20)
Low	8 (27)

a IPAQ SF: International Physical Activity Questionnaire—Short Form.

### Ethical Considerations

All participants provided written informed consent prior to participation in the study. The study protocol was reviewed and approved by the Federal University of Rio Grande do Sul Institutional Review Board (approval 59636722.1.0000.5327). Data collected during the study were anonymized to ensure participant confidentiality, and all privacy measures adhered to institutional and legal requirements. No financial compensation was provided to participants, as participation was entirely voluntary and without any expectation of remuneration.

### Experimental Design

The study was conducted using a crossover design consisting of 3 visits. During the first visit, participants were introduced to the Move Sapiens exergame on the Oculus Quest 2 VR device (Meta) and the 12-exercise HICT. The familiarization session involved participants completing a half session of the exergame, which included 6 blocks of 16 seconds of exercise, followed by 20-second pauses. Additionally, participants performed 10 seconds in each of the HICT exercises. For the randomization of activities, the Research Randomizer tool (Social Psychology Network) [26] was used to generate a random sequence for each participant. This tool is specifically designed for research purposes and provides a reliable method for randomization [26], involved either the Move Sapiens exergame or the control exercise condition (HICT). All sessions occurred within the School of Medicine at Clinics Hospital of Porto Alegre, performed 48 hours apart, and at consistent times of the day to control for circadian variation. On test days, participants were instructed to abstain from other exercises and to avoid alcohol and caffeine for 12 hours before testing. These restrictions were emphasized during the study briefing and reinforced through reminders sent to participants 24 hours before each test session. Compliance was self-reported by participants upon arrival on test days.

Mint chewing gum was provided as a preventive measure to mitigate any potential initial discomfort related to motion sickness during the first visit [27], which served as a familiarization with the VR equipment and protocol. This was intended to help participants acclimate to the VR environment. No formal scale was used during the familiarization session; only anecdotal records about discomfort with VR were kept, and no participants reported any symptoms of motion sickness. Consequently, chewing gum was not provided in subsequent VR sessions, as the initial preventive measure appeared sufficient to alleviate any discomfort during the first exposure.

### Procedures

#### VR Headset and Game Setup

Participants interacted with Move Sapiens using the Oculus Quest 2 VR headset, which was selected because the game was specifically developed for the Oculus platform. The Oculus Quest 2 provides an immersive VR experience through its head-mounted display and 2 handheld controllers, which allow players to engage fully in the game’s mechanics. Before beginning the session, a 5-foot by 5-foot (1.5 m by 1.5 m) play area was calibrated for each participant to ensure safe movement within the virtual environment. The floor level was also adjusted, and the headset straps were customized to fit each participant comfortably, ensuring optimal performance during gameplay.

#### High-Intensity Interval Protocols

In Move Sapiens, players are immersed by VR in a futuristic laboratory while equipped with advanced exoskeleton armor that enhances their physical abilities set within a narrative of futuristic human augmentation via virtual exoskeletons [28]. This setting serves as the backdrop for the first mechanic of the prototype, where the objective is to punch a drone as many times as possible during timed intervals. The armor features a heads-up display that provides players with essential information, including round time, rest periods, the number of completed rounds, and the total punches thrown. Visual and auditory cues guide the players through each phase, ensuring that they follow the high-intensity interval structure. This “hypercasual” prototype focuses on a simple, effective mechanic—delivering rapid punches—while a ranking system tracks the best sessions to motivate continued performance improvement.

We used a low-volume HIIT protocol to mitigate typical VR usability issues such as dizziness [15], in addition to sweat and discomfort [29]. The following short HIIT shadow boxing all-out protocol was used: 12 sets of 16 seconds with 20 seconds of passive rest, lasting approximately 7 minutes, concluding with 1 minute of cooldown [6,23]. The HIIT protocol, including exact times for bouts and rest periods, was custom-integrated into the game. The bouts were controlled by the game itself, which provided cues to start and stop each bout, ensuring that participants followed the protocol accurately. During bouts, players were required to strike the virtual “dummy drone” as many times as possible with punches and scored points each time they hit the target.

**Figure 1.**
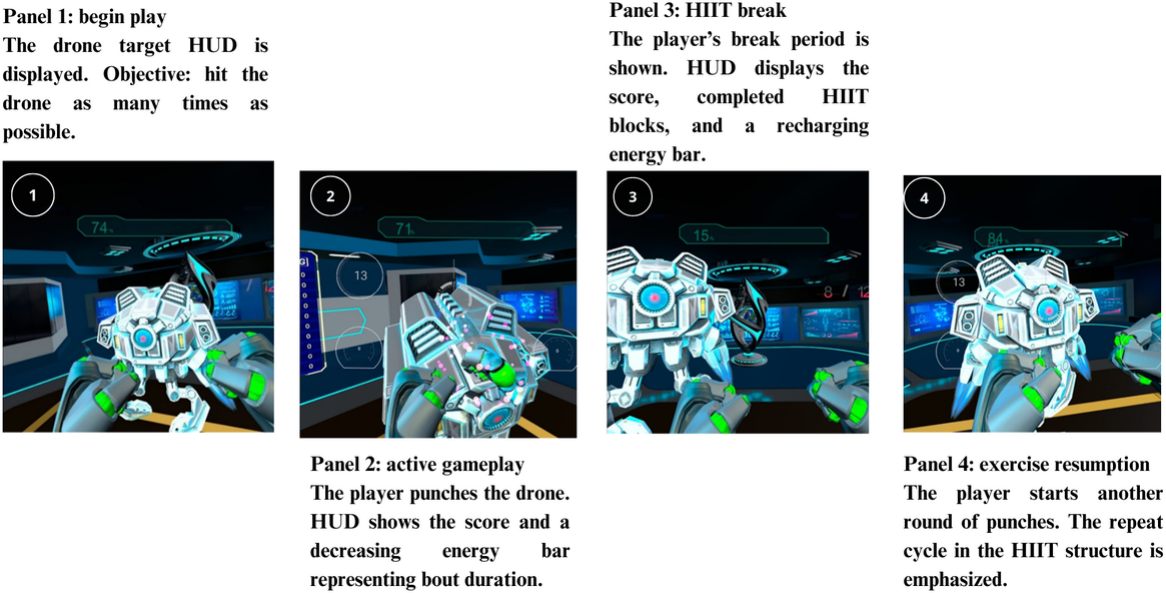
Overview of Move Sapiens HIIT VR exergame mechanics. HIIT: high-intensity interval training; HUD: heads-up display; VR: virtual reality.

The game mechanics are designed to reward the volume and speed of punches, encouraging players to deliver as many blows as possible at a high intensity. This approach motivates players to sustain a high level of effort, resulting in consistently vigorous exercise. In developing the simulation, we focused on creating a fast-punching experience by incorporating essential factors from existing VR boxing games [18,20] but with a more minimalist design [22,30]. This minimalist approach allows players greater freedom in their movements without being constrained by mechanics that require specific, predefined actions [22,31] (Figure 1). The game was developed using the Unreal Engine 4 platform (Epic Games).

As a comparator condition, we used an HICT protocol consisting of 12 exercises including jumping jacks, wall sits, push-ups, abdominal crunches, step-ups onto a chair, squats, triceps dips on a chair, planks, high knees running in place, lunges, push-up and rotation, and side planks [32]. Each exercise was performed for 30 seconds, with 10 seconds of transition time between sets. The total time for the entire circuit training was approximately 7 minutes.

The exercise and rest durations between the HIIT VR protocol and the HICT protocol differ, aimed at assessing external validity. The HICT protocol, with its 7-minute exercise duration, is widely used in home workouts and popularized by smartphone apps, making it a relevant comparison [32]. The objective was to compare 2 existing and widely available do-it-yourself exercise modalities.

### Subjective Measures

After each session, participants completed the Simulator Sickness Questionnaire, which uses a 0‐ to 3-point scale to measure simulator sickness symptoms across nausea, oculomotor, and disorientation subscales. This 16-item questionnaire rates symptoms from 0=none to 3=severe, enabling the evaluation of specific issues like stomach awareness, eyestrain, and dizziness. Responses provide subscale and total scores, quantifying the overall impact of simulator sickness [33]. Subjective perception of effort was recorded at the end of the test using a 6‐ to 20-point RPE scale [34]. The SIMS was used to evaluate both intrinsic and extrinsic motivation among participants, featuring 16 distinct items: (1) I think this activity is interesting, (2) I am doing it for my own good, (3) I am supposed to do it, (4) I do not see any good reasons for doing this activity, (5) I think this activity is pleasant, (6) this activity is good for me, (7) it is something I have to do, (8) I do this activity but do not see its value, (9) this activity is fun, (10) it aligns with how I choose to live my life, (11) I feel obligated to do it, (12) I do this activity but do not know what I gain from it, (13) I enjoy this activity, (14) this activity is important for me, (15) I feel forced to do it, and (16) I do not see what this activity brings me [35]. This comprehensive questionnaire is segmented into 4 motivational factors: intrinsic motivation, identified regulation, external regulation, and amotivation. Each factor is represented by specific items that participants respond to, rating their level of agreement or how applicable each statement feels to them on a nuanced 7-point scale. This scale ranges from 1=does not apply at all to 7=completely applies, facilitating a detailed exploration of participants’ motivational states across various situations. The 16 items are designed to capture a wide range of motivational attitudes, from personal interest and enjoyment (intrinsic motivation) to compliance with external demands (external regulation) and lack of motivation (amotivation).

### Objective Measures

HR was measured continuously during each 7-minute training session using an HR monitor (Polar H10) and was used to calculate average and peak HR. Blood samples were collected from the fingertip and immediately analyzed on a radiometer (ABL 800 flex, Radiometer; Radiometer Medical ApS) to determine blood lactate. Blood collections were performed twice: before the exercise and 5 minutes after the end of the exercise [36].

Data were analyzed using the RStudio software (version 2023.12.1, Build 402; RStudio PBC), and the significance level was defined at *P*<.05. Linear mixed models were used for all the analyses with condition (2 levels: HIIT VR vs HICT) as a fixed factor and participant ID as a random factor. Mean and peak HR as well as postexercise blood lactate were adjusted for baseline values with their addition to the model as a covariate. This was done to account for initial individual differences. This model was used due to its robustness to data missing at random. Data are represented as estimated means (emmeans) and 95% CIs, except stated otherwise. Cohen *d* was used to calculate effect sizes for objective and subjective data, offering a standardized way to evaluate the practical significance of the observed effects. For Cohen *d* calculation, a transformation from Student *t* values to Cohen *d* was performed:


$$d=2*t\;÷\;\sqrt{df_{error}}$$

Cohen *d* classifications are interpreted as follows: a value below 0.2 indicates a very small effect, above 0.2 indicates a small effect, above 0.5 indicates a medium effect, and above 0.8 indicates a large effect.

## Results

**Figure 2.**
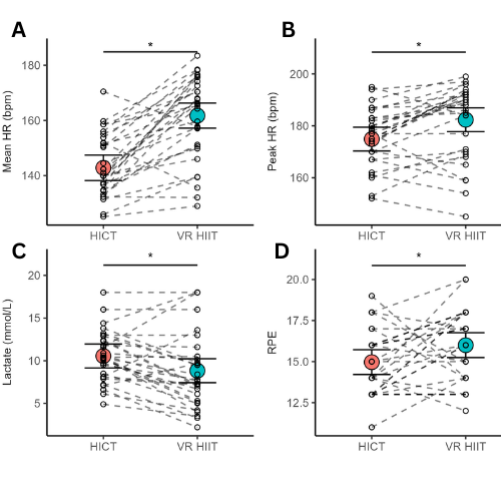
Mean values of (A) mean HR, (B) peak HR, (C) postexercise lactate, and (D) RPE, along with their respective 95% CIs for the standard HIIT protocol (pink circles) and VR HIIT (blue circles). Individual participant data are represented by small dots, while a line connects their values between conditions. HICT: high-intensity circuit training; HIIT: high-intensity interval training; HR: heart rate; RPE: ratings of perceived exertion; VR: virtual reality. *Significant differences between protocols.

The results revealed significant differences in several metrics between VR HIIT and HICT. For mean HR, VR HIIT exhibited higher values (emmean 162 bpm, 95% CI 157-166) compared to HICT (emmean 143 bpm, 95% CI 138-147; *P*<.001; *d*=2.07; 95% CI 1.40-2.72; Figure 2A). Peak HR was also higher in VR HIIT (emmean 182 bpm, 95% CI 178-187) compared to HICT (emmean 175 bpm, 95% CI 170-180; *P*=.001; *d*=1.20; 95% CI 0.61-1.77; Figure 2B). Postexercise lactate concentration was higher following HICT (emmean 10.6 mmol/L, 95% CI 9.14-12.0) compared to VR HIIT (emmean 8.83 mmol/L, 95% CI 7.43-10.2; *P*=.006; *d*=0.80; 95% CI 0.25-1.34; Figure 2C). RPE were higher for VR HIIT (emmean 16, 95% CI 15-17) compared to HICT (emmean 15, 95% CI 14-16; *P*=.03; *d*=0.61; 95% CI 0.07-1.14).

**Figure 3.**
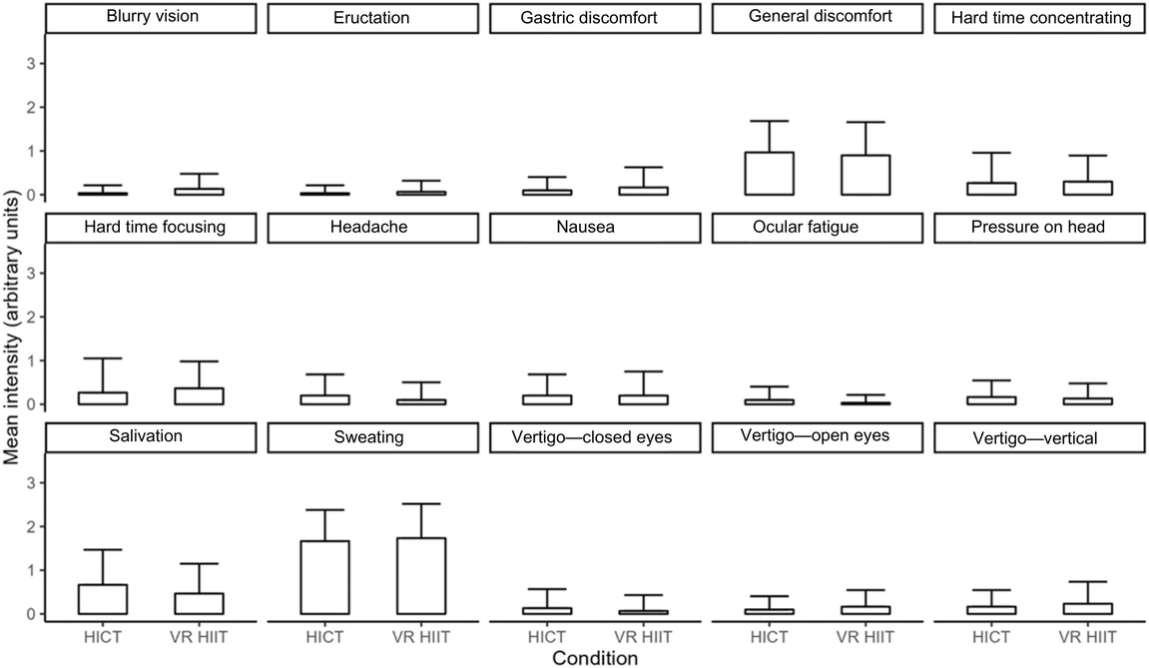
Summary of mean values for symptoms as reported by the participants. Values are represented as mean (SD). HICT: high-intensity circuit training; HIIT: high-intensity interval training; VR: virtual reality.

Regarding reported symptoms, perceived fatigue (not RPE) was higher for VR HIIT (emmean 1.8, 95% CI 1.6-2.0) compared to HICT (emmean 1.5, 95% CI 1.3-1.7; *P*=.02; *d*=0.67; 95% CI 0.13-1.20). No other differences in symptoms were observed between conditions (all *P*≥.14; Figure 3). There were no significant differences between VR HIIT and HICT for intrinsic motivation (*P*=.06; *d*=0.53), identified regulation (*P*=.70; *d*=0.10), external regulation (*P*=.10; *d*=0.32), or amotivation (*P*=.35; *d*=0.26).

## Discussion

### Principal Findings

The findings from this study underscore the efficacy of VR HIIT exergaming to increase physiological measures such as HR and increase perceived exertion without reducing intrinsic motivation when compared to traditional HICT. This demonstrates that VR HIIT is not just effective in providing physical exercise benefits akin to those of HICT but also has the potential to maintain acute motivation for exercise. Despite the greater perceived exercise intensity, intrinsic motivation did not decrease, given the sensory stimuli of the VR environment. The immersive nature of VR HIIT, marked by interactive and engaging elements, may contribute to these positive outcomes [21,22]. This aligns with previous studies showing that VR exergaming effectively increases enjoyment during a single bout of HIIT in untrained individuals [14] and improves mood [16].

Lactate levels were substantially increased following both exercise sessions but were significantly higher in the HICT condition compared to the VR HIIT. This can be attributed to the nature of HICT, which typically incorporates a higher volume of strength exercises and calisthenics using body weight [32]. Such activities are known to facilitate a greater accumulation of metabolites due to the anaerobic nature of the exertion, leading to higher lactate production [37]. Despite this, the VR HIIT condition also achieved substantial blood lactate concentrations, indicative of significant metabolic stress [23,38]. Notably, this was achieved alongside higher values in HR during the exercise, suggesting that this VR HIIT boxing protocol effectively stimulates cardiovascular and metabolic responses even in the absence of traditional strength and calisthenic exercises [8]. Our HR results demonstrated that the VR HIIT boxing protocol achieved vigorous intensity levels comparable to the “Supernatural” VR fitness game’s flow and boxing modes. These modes are associated with significant caloric expenditure, ranging from approximately 12.01 to 13.11 kilocalories per minute, with metabolic equivalent of task values of 11.44 for flow and a peak of 12.49 for boxing at higher intensities. [20].

Importantly, the VR game, although a distinct exercise modality from HICT, induced metabolic stress like that of an exercise model validated to be performed independently [32]. This finding underscores the potential of VR HIIT to offer a comparable physiological challenge to HICT, leveraging the immersive and engaging qualities of VR technology to simulate a validated exercise environment effectively.

The exercise intensity within the VR setting is inherently self-selected, despite the game design being crafted to encourage engagement at the highest possible intensity levels [9,21]. Similarly, exercise intensity is also self-selected for HICT, as every type of exercise involves motivation, volition, and intensity self-regulation. The incentive to increase intensity in the game was a better score achieved via the greatest number of punches within the HIIT blocks [21,31]. The stimulus of the VR game may have led to increased effort during the activity, leading to increased HR. Whether this leads to greater health benefits or increased engagement in exercise over time remains to be investigated. Evidently, how to increase motivation response in VR is a considerable question ahead [39]. The foremost challenge lies in the evolution of game design, where the objective is to increasingly leverage game mechanics and sensory stimuli to foster higher motivation among users [21,22]. However, it is important to acknowledge that the novelty of VR may initially boost motivation and engagement, potentially skewing performance in the short term [40]. This effect underscores the need for creating more compelling and immersive experiences that not only draw participants in [39] but also encourage them to exert themselves more vigorously during the exercise [9,14]. This endeavor requires a nuanced understanding of human motivation and behavior, alongside a mastery of VR technology, to craft experiences that are both engaging and physically demanding [41].

### Future Directions

Integrating HIIT with VR in the study demonstrated a notable safety profile, with no adverse effects reported, particularly concerning motion sickness. The intersection of HIIT and VR with continuous innovation [42] represents a promising approach, particularly for improving engagement and motivation in exercise routines [21,43]. By combining the cognitive and physiological benefits of HIIT with the immersive qualities of VR, this approach holds the potential for a holistic method that addresses physical health. While this study did not specifically examine mental health special populations, the immersive and engaging nature of VR HIIT suggests potential applicability for these groups. This is particularly relevant for youth populations, such as patients with attention-deficit/hyperactivity disorder [44,45], who may benefit from such a different and multifaceted approach to exercise [21,46]. While the study confirmed safety among 30 medical students with heterogeneous physical activity levels, the findings are specific to this group. More research is needed to determine the effectiveness of VR HIIT exergames for these kinds of patients.

### Limitations

This study acknowledges certain limitations, primarily its reliance on physiological markers, such as HR and blood lactate levels, and subjective motivation assessments [47]. The study uniformly administered the Simulator Sickness Questionnaire after the exercise across both experimental and control conditions. While this approach maintains comparability, it does not capture baseline symptom levels [48]. We chose to compare an HIIT VR protocol to a traditional HICT protocol, which may have led to some of the differences observed.

The study uniformly administered the SIMS after the exercise across both experimental and control conditions. While this approach maintains comparability, it does not capture baseline motivation or symptom levels [48], limiting our ability to assess changes in motivation due to the exercise protocols themselves. Additionally, the choice of highly active participants, who likely had high baseline motivation, may have introduced a ceiling effect, making it difficult to detect significant changes in motivation between the VR HIIT and HICT conditions. This limitation should be considered when interpreting the null findings in motivation, as the participants’ pre-existing motivation levels could have constrained the potential for further increases. Importantly, we do not infer any superiority of either exercise type despite some differences in physiological responses, and further research should make comparisons between our VR HIIT protocol and other non-VR HIIT protocols.

The data derived from this study do not necessarily suggest that VR HIIT will be better adhered to over the long term nor that it will generate similar or better results when applied in a prolonged context. This points to a significant area for future investigation, emphasizing the need to assess the long-term adherence to, and effectiveness of, VR HIIT programs [16,39]. Despite these constraints, the findings contribute valuable information on the physiological responses to VR HIIT. Although the study did not show significant changes in intrinsic motivation, it demonstrated that VR HIIT does not reduce intrinsic motivation compared to HICT. Since subjective motivation plays a crucial role in determining whether participants will continue to engage in an activity [39,49], future research should aim to enhance subjective motivation and examine its impact on long-term adherence and engagement.

### Conclusions

VR HIIT achieves acute significant increases in key physiological measures, affirming its effectiveness as an exercise modality comparable to traditional HICT in terms of likely long-term physical benefits. The combination of VR and HIIT has proven to be safe, with no adverse effects, and has maintained intrinsic motivation despite greater perceived exercise intensity due to the sensory stimuli provided by the VR environment. Additionally, VR HIIT’s capacity to deliver immersive and tailored exercise experiences presents promising applications in therapeutic contexts, particularly for populations with specific needs where conventional exercise methods may fall short. While this study demonstrates the immediate benefits of VR HIIT, future research is essential to evaluate the sustained engagement and long-term health outcomes associated with this modality. Investigating its impact over extended periods will be crucial to fully understand the breadth of VR HIIT’s benefits and to optimize its application for various exercise and therapeutic needs, particularly in clinical populations.

## Abbreviations

emmean: estimated mean
HICT: high-intensity circuit training
HIIT: high-intensity interval training
HR: heart rate
RPE: ratings of perceived exertion
SIMS: Situational Motivation Scale
VR: virtual reality
